# Inhibition of Synovial Macrophage Pyroptosis Alleviates Synovitis and Fibrosis in Knee Osteoarthritis

**DOI:** 10.1155/2019/2165918

**Published:** 2019-09-08

**Authors:** Li Zhang, Runlin Xing, Zhengquan Huang, Nongshan Zhang, Li Zhang, Xiaochen Li, Peimin Wang

**Affiliations:** ^1^The Affiliated Hospital of Nanjing University of Chinese Medicine, Department of Orthopedics, Nanjing, China; ^2^Hospital of Nanjing University of Chinese Medicine, No. 155 Hanzhong Road, Nanjing, Jiangsu Province, China

## Abstract

Increasing evidence has shown that macrophage pyroptosis in different tissues participates in chronic aseptic inflammation and is related to tissue fibrosis. Our last studies also revealed the vital role of synovial fibroblast pyroptosis in the onset and development of knee osteoarthritis (KOA). In this study, we aimed to investigate whether synovial macrophage pyroptosis did occur and whether this form of cell death should be related to synovitis and fibrosis of KOA. In the synovial tissue of KOA model rats, we observed a decrease of caspase1, NLRP3, ASC, and GSDMD caused by macrophage depletion in both the mRNA and protein expressions. Besides, rats treated with the specific caspase1 inhibitor Ac-YVAD-CMK showed less inflammatory reaction and fibrosis, not only in the expression of proinflammatory factors IL-1*β*, IL-18, and HMGB1 and fibrosis markers TGF-*β*, PLOD2, COL1A1, and TIMP1 but also in the observation of HE staining, Sirius Red staining, and the transverse diameters of the right knees. Subsequently, we established an LPS+ATP-induced model in macrophages mimicking the inflammatory environment of KOA and inducing macrophage pyroptosis. Macrophages transfected with caspase1 siRNA showed reduced cell death; meanwhile, the relative expression of pyroptosis-related proteins were also downregulated. In addition, the level of fibrotic markers in synovial fibroblasts were significantly decreased after coculture with siRNA GSDMD-transfected macrophages. To conclude, synovial macrophage pyroptosis may occur in the pathological processes of KOA and inhibition of synovial macrophage pyroptosis alleviates synovitis and fibrosis in KOA model rats.

## 1. Introduction

Knee osteoarthritis (KOA) is a degenerative joint disorder that affects all tissues in the knees, while chronic low-grade inflammation is a major driver of the ongoing joint degeneration [1]. Symptomatically, KOA is characterized by the progressive destruction of articular cartilage and surrounding tissues, especially synovial tissue, causing pain, stiffness, and disability [2]. Synovial tissue is the soft membrane lining the spaces of knees and contains highly metabolically active cells called synoviocytes which are further separated as synovial macrophages and synovial fibroblasts. These cell types and the synovial tissue as a whole play a key role in normal joint physiology as they facilitate nourishment for chondrocytes and remove the waste products through a synovial fluid [3]. So far, we lack a comprehensive understanding of the pathogenesis of KOA, and the present study generally agrees that synovitis plays an important role in the development of KOA.

The infiltration and activation of macrophages in synovial tissue make synovitis a primary trigger of KOA's pathogenesis. Its importance has been implicated by their close relevance to KOA symptoms such as cartilage destruction and synovial fibrosis [4]. The fact that macrophages are involved in KOA even makes them valid candidates to use in the quantitative assessment of inflammation [5]. Synovial fibrosis is another pathological change of synovial tissue characterized by excessive extracellular matrix deposition, which contributes to joint pain and stiffness. Genes procollagen-lysine, 2-oxoglutarate 5-dioxygenase 2 (PLOD2), collagen type I *α*1 chain (COL1A1), and tissue inhibitor of metalloproteinase 1 (TIMP1) are shown upregulating in osteoarthritis-related fibrosis; they are usually considered to be fibrotic markers [6]. Besides, accumulating evidence has demonstrated the critical role of synovial fibroblasts and transforming growth factor-*β* (TGF-*β*) in fibrotic response [7, 8].

Recently, a new form of programmed cell death, mostly found in monocytes, macrophages, and dendritic cells, has been described as pyroptosis [9]. In the canonical pathway of pyroptosis, NLRP3, the most well-studied nod-like receptor, forms an inflammasome comprised of apoptosis-associated speck-like protein (ASC) and the serine protease caspase1 [10]. NLRP3 inflammasome activation governs the cleavage and activation of pro-caspase1. Finally, activated caspase1 causes proinflammatory cytokines such as pro-IL-1*β*, pro-IL-18, and HMGB1 to mature and induces pyroptosis depending on the cleavage of gasdermin D (GSDMD) [11, 12]. In our last studies, the correlation between the NLRP inflammasomes and synovial fibroblast pyroptosis was investigated in vivo and in vitro [13]. We also revealed that increased hypoxia-inducible factor-1*α* in KOA aggravates synovial fibrosis via synovial fibroblast pyroptosis [14].

A study on macrophage pyroptosis is initially concentrated on infection caused by bacteria and viruses [15]. But increasing evidence has shown that macrophage pyroptosis in different tissues participates in chronic aseptic inflammation and is related to tissue fibrosis. For instance, microglia (i.e., macrophages of the central nervous system) pyroptosis can induce neurogenic inflammation and fibrosis [16], liver Kupffer cell (i.e., macrophages in liver tissue) pyroptosis can cause chronic hepatitis and liver fibrosis [17], and macrophage pyroptosis in the kidney can lead to renal inflammation and fibrosis [18].

In summary, KOA shows a state of low-grade inflammation and a combination of synovitis and fibrosis. These pathological processes are remarkably similar to those “macrophage pyroptosis/inflammation/fibrosis” in the field of other tissues. Accordingly, we hypothesized that there should be macrophage pyroptosis in synovial tissues and this form of cell death should be related to synovitis and fibrosis.

## 2. Materials and Methods

### 2.1. In Vivo Animal Experimental Design

Thirty-two 3-month-old SD male rats, with weights ranging from 250 g to 290 g (provided by Beijing Vital River Laboratory Animal Technology Co. Ltd.), were used. Animals were maintained in a specific pathogen-free, laminar-flow housing apparatus under controlled temperature, humidity, and 12 h light/dark regimen. All animal protocols were approved by the Animal Care and Use Committee of the Nanjing University of Chinese Medicine. All experiments were conducted in accordance with the National Institutes of Health Guidelines for the Care and Use of Laboratory Animals.

Rats were randomly assigned to the following four groups (with eight rats in each group): Normal, KOA, KOA+Clodronate Liposomes (KOA+CL), and KOA+Ac-YVAD-CMK (KOA+AYC). On Day 1, the KOA model was constructed by anterior cruciate ligament transection (ACLT) surgery in both knees as described previously [19]. 14 days after surgery (Day 14), drug administration began. The KOA+CL group was depleted of synovial lining macrophages by intra-articular injection of 100 *μ*l Clodronate Liposomes (Clodronate Liposomes, Amsterdam, Netherlands) [20]. The KOA+AYC group was intra-articularly injected with caspase1 inhibitor Ac-YVAD-CMK (Sigma-Aldrich, St. Louis, MO, USA) at a dose of 0.25 mg/kg (in saline with 10% DMSO, 100 *μ*l). The Normal group and the KOA group were treated with 100 *μ*l sterilized physiologic saline at the same time. All drugs were injected every 2 days for 2 weeks. After that, rats were sacrificed to harvest the synovial tissue.

### 2.2. Measurement of the Right Knee Joint Diameter

The transverse diameters of the right knees were measured with a slide caliper at Day 1, Day 7, Day 14, Day 21, and Day 28 (Mitutoyo, Kanagawa, Japan) to measure the horizontal distance between the left and right highest points of the knee joints flexed at 90°, respectively. Each knee was measured three times, and the mean value was calculated.

### 2.3. Immunofluorescence Assay

The synovial tissues were frozen for the preparation of sections. Sections were washed in PBS for 5 min and processed in 0.3% H_2_O_2_ in methanol for 20 minutes. The goat serum was added to the block for 30 min at room temperature. Then, the sections were incubated with primary antibodies of anti-CD68 (a panmacrophage marker [21], Abcam, Cambridge, UK) at 4°C overnight and with secondary antibodies of anti-goat/rabbit at 37°C for 2 h. CD68 was stained with bright orange fluorescence, and sections were observed under a fluorescence microscope (Zeiss, Germany).

### 2.4. Histological Analysis

Synovial tissues were collected after executing rats, and tissues were then fixed in 10% neutral formalin, embedded in paraffin, and cut into slices for routine HE staining. Sirius Red staining was carried out according to the instructions of the Sirius Red Stain Kit (Beyotime Biotechnology, Shanghai, China). Briefly, the tissue sections were dewaxed, dipped into water, stained with 1 g/l picric acid-Sirius Red at 37°C for 1 h, and then washed with water. The sections were mounted and viewed under a Leica DMI3000B microscope (Leica, Germany) with the use of a bright field.

### 2.5. Cell Preparation

Primary rat synovial fibroblasts and macrophages were obtained from additional normal rats. In brief, synovial tissues were washed for 2-3 times with phosphate-buffered saline (PBS) and then minced into pieces of 2-3 mm^2^ and digested in 0.1% collagenase type II (Sigma-Aldrich, St. Louis, MO, USA) for 30 min. Following cell dissociation, the samples were filtered through a cell strainer. After dissociation, fibroblasts were pelleted by centrifugation at 1500 rpm for 4 min and cultured in DMEM supplemented with 10% fetal bovine serum (FBS; Gibco, Thermo Fisher Scientific, Waltham, MA, USA) and antibiotics (100 U/ml penicillin, 100 *μ*g/ml streptomycin; Invitrogen, CA, USA). Macrophages were obtained from the peritoneal exudates with PBS containing 2% FBS. Cells were identified as our previous studies [15]. Passages 3-6 of the synovial fibroblasts were used for the experiments.

### 2.6. Small Interfering RNA Preparation and Transfection

To inhibit the caspase1 and GSDMD expression in the macrophages, commercially available caspase1, GSDMD, and vehicle/scrambled small interfering RNA (Invitrogen, CA, USA) were used. Macrophages were transfected with siRNAs by using Lipofectamine 2000 (Invitrogen, CA, USA) according to the manufacturer's instructions. siRNA was diluted in a transfection reagent and culture medium, and the cells were incubated with 20 pmol siRNA for 6 h.

### 2.7. Cell Treatment

LPS+ATP were used to simulate the inflammatory environment of KOA and induce cell pyroptosis. Macrophages transfected with caspase1 siRNA (Caspase1 siRNA group) or vehicle/scrambled siRNA (KOA group) were stimulated with LPS (1 *μ*g/ml) in DMEM for 6 h and then challenged with ATP (3 mM) for 1 h. The macrophages exposed to DMEM with the same volume of PBS served as controls (Normal group).

To evaluate the relationship between pyroptosis and fibrosis, macrophages were plated into 6-well plates. The siRNA GSDMD group, the KOA group, and the Normal group were transfected with siRNA and stimulated with LPS as described above. After induction, macrophages were cultured in DMEM supplemented with 10% FBS. Then, synovial fibroblasts precultured in a Transwell chamber were placed on a 6-well plate and cocultured with macrophages for 48 hours.

### 2.8. Hoechst and Propidium Iodide (PI) Staining

To evaluate pore formation in the cell membrane, Hoechst 33342 and PI (Beyotime Biotechnology, Shanghai, China) were used for staining. Briefly, macrophages were stained with Hoechst for 10 min. After washing three times with phosphate-buffered saline, PI was added for another 10 min. Cells were observed using a fluorescence microscope (Leica DMI3000B, Germany).

### 2.9. Western Blotting

Briefly, synovial tissues or cells were mixed with RIPA lysate and ground for 10-15 min. Samples were agitated on ice for 30 min and then the supernatant was collected. The protein levels were quantified with a BCA protein assay kit (Beyotime Biotechnology, Shanghai, China). Then, the protein samples were electrophoresed in SD-PAGE to separate protein bands. Proteins were transferred from the gel onto the PVDF membrane and blocked with 5% nonfat dry milk for 2 h. The membrane was incubated with a primary antibody (1 : 1000; Abcam, Cambridge, UK) overnight at 4°C and then with a secondary antibody (Thermo Fisher Scientific, Shanghai, China) for 2 h. Later, bands were visualized by exposure to the ECL method and the overall gray value of the protein bands (average gray value/gray value area) was quantified; GAPDH was used as an internal marker, namely, target protein gray value/internal reference overall gray value.

### 2.10. Real-Time PCR

Briefly, total RNA was extracted with TRIzol and assessed by a spectrophotometer. Then, RNA from each group and 10 *μ*l PCR reaction solution were added into 10 *μ*l of the Prime Script RT Reagent Kit (Beyotime Biotechnology, Shanghai, China) for reverse transcription at conditions of 37°C (15 min) and 85°C (5 s). The primer was designed and synthesized by Shanghai Biotechnology Service Company in accordance with the gene sequence in the GenBank gene sequence design, together with OLIGO v6.6. Sequences are shown in Table 1. qPCR was performed using Premix Ex Taq SYBR Green PCR (TaKaRa) according to the manufacturer's instructions on an ABI PRISM 7300 (Applied Biosystems, Foster City, CA, USA). The mRNA level of individual genes was normalized to GAPDH and calculated by the 2^−ΔΔCT^ data analysis method.

### 2.11. Statistical Analysis

Statistical analysis was performed using GraphPad Prism 6.0 Software (San Diego, CA, USA). Data are presented as mean ± standard deviation (SD). Group comparisons were assessed with one-way ANOVA or Student's *t*-test or two-way ANOVA with Bonferroni's post hoc test for comparison of multiple columns. A value of *P* < 0.05 (two-tailed) was considered as statistically significant.

## 3. Results

### 3.1. Synovial Macrophage Pyroptosis in KOA Model Rats

**Figure 1:**
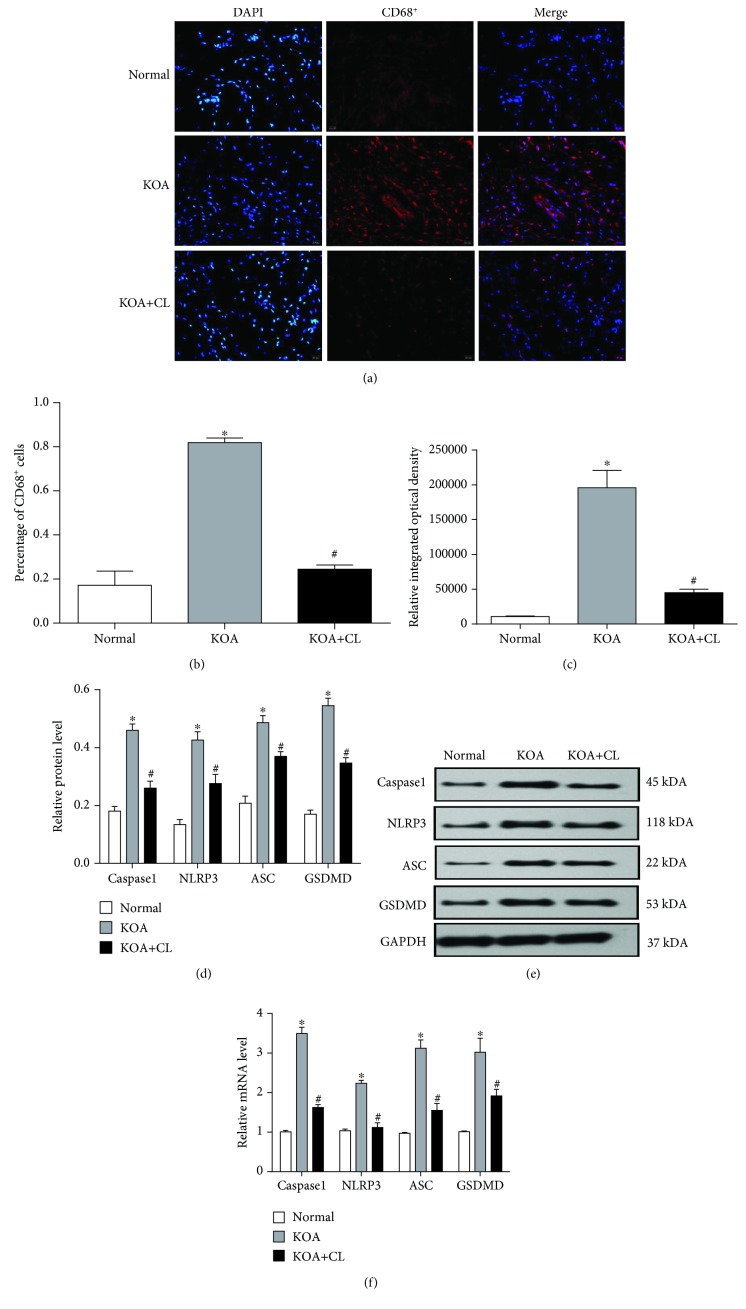
Synovial macrophage pyroptosis in KOA model rats. (a) Representative synovial tissues stained with DAPI and TRITC. Scale bar = 20 *μ*m (×100). (b) Percentage of CD68^+^ cells in each group. ^∗^*P* < 0.05, in comparison with the Normal group. ^#^*P* < 0.05, in comparison with the KOA group. (c) Relative integrated optical density (IOD) value in each group. Data were analyzed by ImageJ. (d) Protein expressions of caspase1, NLRP3, ASC, and GSDMD between groups. ^∗^*P* < 0.05, in comparison with the Normal group. ^#^*P* < 0.05, in comparison with the KOA group (*n* = 6). Western blot data are presented from three independent experiments. (e) Typical protein bands for each group. (f) mRNA level of caspase1, NLRP3, ASC, and GSDMD between groups. Trend is consistent with protein expression.

An immunofluorescence assay (Figure 1(a)) was performed to confirm that synovial macrophages had been depleted. The KOA group resulted in a significant upregulation in the percentage of CD68^+^ cells (Figure 1(b)) compared with the Normal group. Meanwhile, the KOA+CL group showed a downregulation compared with the KOA group that suggests a depletion of macrophages in the KOA+CL group. Comparison of the relative integrated optical density (IOD) value in each group was consistent with the variation tendency of CD68^+^ cells (Figure 1(c)). Subsequently, we analyzed both gene and protein expressions of caspase1, NLRP3, and ASC, which constitute the NLRP3 inflammasome. Our data showed that the KOA group resulted in a significant upregulation (Figures 1(d)–1(f)) compared with the Normal group, and the KOA+CL group showed a downregulation compared with the KOA group. In addition, the GSDMD level showed the same trend.

### 3.2. Inhibition of Synovial Macrophage Pyroptosis May Alleviate Synovitis in KOA Rats

**Figure 2:**
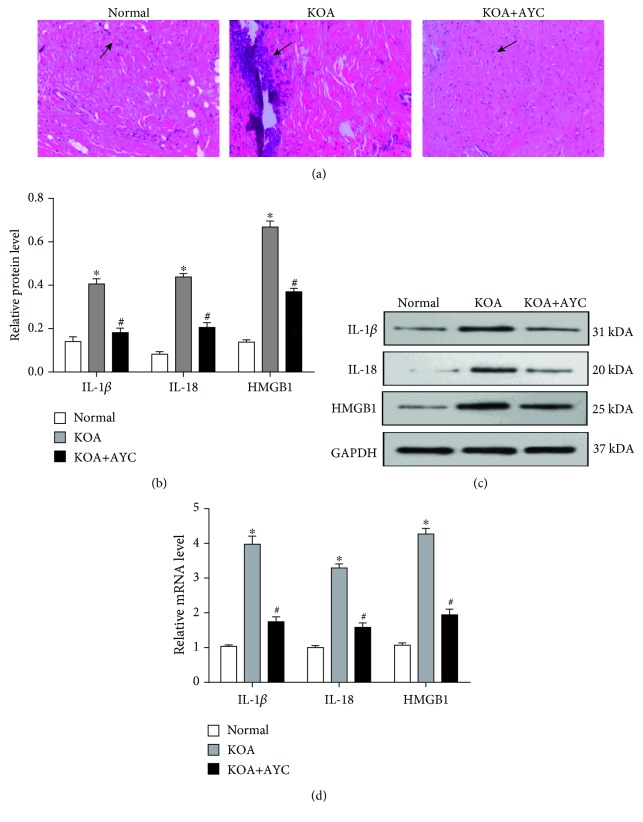
Inhibition of synovial macrophage pyroptosis may alleviate synovitis in KOA rats. (a) Representative synovial tissues of each group stained with HE staining (200×). Arrow: inflammatory cell infiltration. (b) Protein expressions of IL-1*β*, IL-18, and HMGB1 between groups. ^∗^*P* < 0.05, in comparison with the Normal group. ^#^*P* < 0.05, in comparison with the KOA group (*n* = 6). (c) Typical protein bands for each group. (d) mRNA levels of IL-1*β*, IL-18, and HMGB1 between groups. The trend is consistent with protein expression.

In order to evaluate synovitis, hematoxylin and eosin staining was performed. Compared with the KOA group, the KOA+AYC group showed an orderly arranged lining of synovial cells, loose connective tissue, and less inflammatory cell infiltration (Figure 2(a)). In addition, both mRNA and protein expressions of IL-1*β*, IL-18, and HMGB1 were measured (Figures 2(b)–2(d)). We found that there was a significant decrease in the expression of these proinflammatory cytokines in the KOA+AYC group.

### 3.3. Inhibition of Synovial Macrophage Pyroptosis May Alleviate Fibrosis in KOA Rats

**Figure 3:**
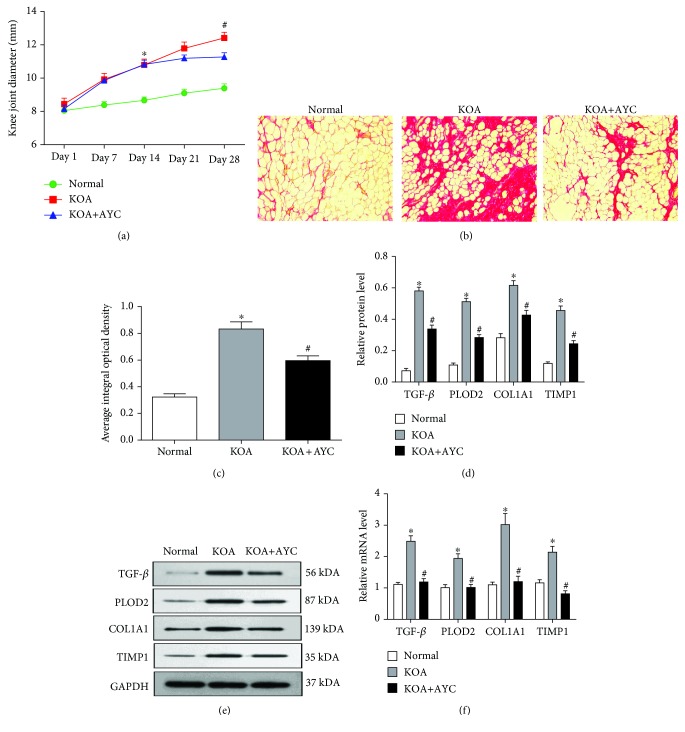
Inhibition of synovial macrophage pyroptosis may alleviate fibrosis in KOA rats. (a) The severity of synovial fibrosis evaluated by the transverse diameters of the right knees. ^∗^*P* < 0.05 vs. the Normal group; ^#^*P* < 0.05 vs. the KOA+AYC group. (b) Collagen deposition was revealed through Sirius Red staining (200×). (c) Semiquantification of Sirius Red staining was evaluated using the average integral optical density (AIOD). The results were expressed as the mean ± SD (*n* = 6). ^∗^*P* < 0.05, in comparison with the Normal group. ^#^*P* < 0.05, in comparison with the KOA group. Data were analyzed by ImageJ. (d) Comparison of TGF-*β*, PLOD2, COL1A1, and TIMP1 protein expressions between groups. ^∗^*P* < 0.05, in comparison with the Normal group. ^#^*P* < 0.05, in comparison with the KOA group (*n* = 6). (e) Typical protein bands for each group. (f) The mRNA levels of TGF-*β*, PLOD2, COL1A1, and TIMP1 between groups.

**Figure 4:**
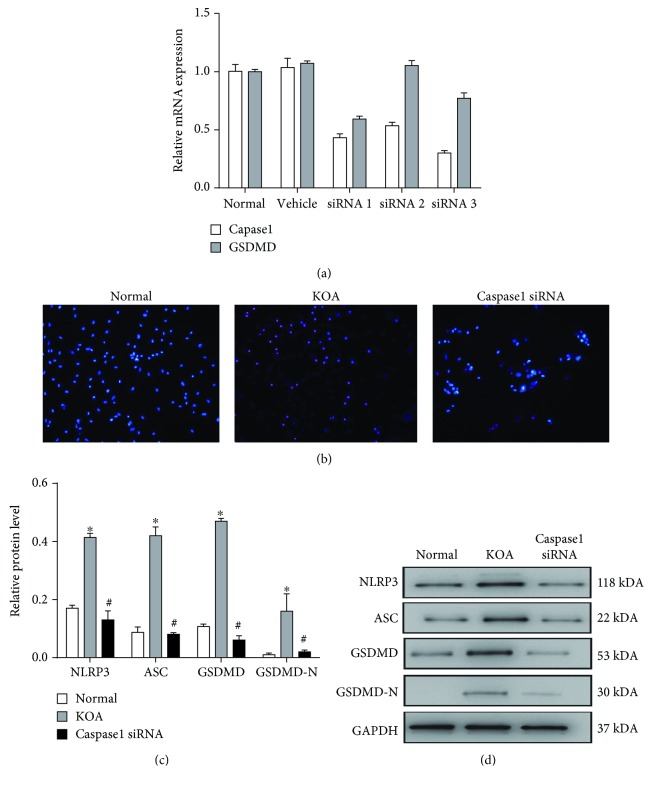
Pyroptosis is induced in LPS+ATP-treated macrophages. (a) The silencing effects of the caspase1 siRNA and GSDMD siRNA were both confirmed by PCR. (b) Pyroptotic cells observed by Hoechst and PI staining (200×). (c) Comparison of NLRP3, ASC, GSDMD, and GSDMD-N protein expressions between groups. ^∗^*P* < 0.05, in comparison with the Normal group. ^#^*P* < 0.05, in comparison with the KOA group. (d) Typical protein bands for each group.

To quantify the degree of knee swelling in rats, transverse diameters of the right knees were measured (Figure 3(a)). On Day 14, the knee joint diameter of the KOA group was significantly larger than that of the Normal group, and on Day 28, the knee joint diameter of the KOA+AYC group was significantly smaller than that of the KOA group. To observe the fibrosis of synovial tissue, we carried out Sirius Red staining. Compared with the Normal group, the KOA group showed markedly increased collagen deposition. This change was alleviated in the KOA+AYC group (Figure 3(b)). Semiquantification of Sirius Red staining (Figure 3(c)) was evaluated using the average integral optical density (AIOD). The KOA+AYC group showed a significant downregulation compared with the KOA group. Furthermore, we analyzed mRNA and protein expressions of TGF-*β*, PLOD2, COL1A1, and TIMP1 (Figures 4(d)–4(f)). We found that there was a significant decrease in the level of these profibrotic substances in the KOA+AYC group.

### 3.4. Pyroptosis Is Induced in LPS+ATP-Treated Macrophages

An LPS+ATP-induced model in macrophages was established mimicking the inflammatory environment of KOA and inducing macrophage pyroptosis. The silencing effect of the caspase1 siRNA and GSDMD siRNA was confirmed by PCR (Figure 4(a)). Hoechst and PI staining showed that the number of pyroptotic cells was increased by LPS+ATP treatment, and this trend can be reversed by caspase1 siRNA (Figure 4(b)). Besides, the protein expressions of NLRP3, ASC, GSDMD, and cleavage-GSDMD (GSDMD-N) in the KOA group were higher than those in the Normal group, and the Caspase1 siRNA group showed a significant decrease of these key pyroptosis-related substances compared with the KOA group (Figures 4(c) and 4(d)).

### 3.5. Macrophage Pyroptosis Upregulates the Expression of Fibrosis Markers Cocultured with Synovial Fibroblasts

**Figure 5:**
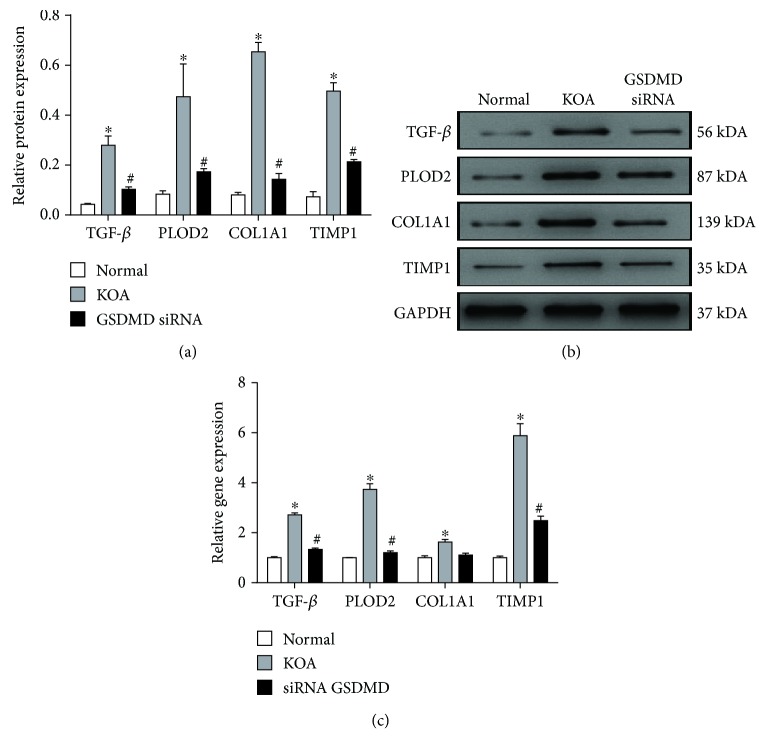
Macrophage pyroptosis upregulates the expression of fibrosis markers cocultured with synovial fibroblasts. (a) Comparison of TGF-*β*, PLOD2, COL1A1, and TIMP1 protein expressions between groups. ^∗^*P* < 0.05, in comparison with the Normal group. ^#^*P* < 0.05, in comparison with the KOA group. (b) Typical protein bands for each group. (c) Relative mRNA expression of TGF-*β*, COL1A1, PLOD2, and TIMP1 in synovial fibroblasts in each group. ^∗^*P* < 0.05, in comparison with the Normal group. ^#^*P* < 0.05, in comparison with the KOA group.

After coculture for 48 hours, both mRNA and protein expressions of TGF-*β*, PLOD2, COL1A1, and TIMP1 in synovial fibroblasts were detected. These fibrosis markers showed a significant upregulation in the KOA group compared with the Normal group, and a downregulation compared with the siRNA GSDMD group (Figures 5(a)–5(c)).

## 4. Discussions

The current study indicates that synovial macrophage pyroptosis participates in the pathologic process of KOA. Inhibition of synovial macrophage pyroptosis alleviates synovitis and fibrosis in KOA. ACLT surgery was performed to construct KOA model rats. ACLT is modified from the Hulth method and widely used for modeling KOA. ACLT results in joint instability and thus induces cartilage degeneration, subchondral bone sclerosis, and osteophyte formation, which mimics the pathological changes observed in human OA [22]. This method has less trauma and physiological structure changes than the Hulth method and develops less rapidly compared with MIA method [23]. Therefore, it is more suitable for this study.

We revealed that pyroptosis occurs in synovial macrophages by comparing synovial tissue with or without the depletion of synovial macrophages. We also conducted a series of quantitative studies to demonstrate that synovial macrophage pyroptosis is related to synovitis and fibrosis in vivo. In addition, we found the high expression of fibrosis markers induced by pyroptosis through macrophages and synovial fibroblasts cocultured in vitro. Since the symptoms of KOA are directly related to the severity of synovitis and fibrosis, the findings of this paper may suggest a novel role for synovial macrophages in the pathogenesis of KOA.

Macrophages play a crucial role in the progression of KOA and synovial macrophage activity, and their mediators have been regarded as potential therapeutic targets in OA. While exposed to inflammatory conditions, macrophages can become activated and may acquire a phenotype, ranging from proinflammatory (M1) to anti-inflammatory (M2) phenotypes [24]. Utomo et al. proved that macrophage phenotype modulation can be used to guide joint inflammation. Furthermore, the team also proved that cartilage inflammation and degeneration could be enhanced by M1 macrophages via the upregulation of IL-1*β*, IL-6, MMP-13, and ADAMTS-5 [25, 26]. Increasing evidence has shown that macrophage pyroptosis in different tissues such as liver, renal, and airway tissues participates in chronic aseptic inflammation and can be related to tissue fibrosis. It is also widely recognized that the cleavage of GSDMD plays a crucial role in pyroptosis [16–18]. In the canonical pathway, pyroptosis may require a two-step mechanism. At the first stage, proinflammatory mediators pro-IL-1*β* and pro-IL-18, caspase1, and NLRP family members are transcriptionally generated. The second stage is activation, including the NLRP inflammasomes assembling and caspase1 activating. Activated caspase1 further causes pro-IL-1*β* and pro-IL-18 to mature and induces pyroptosis depending on the cleavage of GSDMD [11]. Unlike the canonical pathway, caspase1 can be activated by directly sensing intracellular LPS and by promoting pyroptosis through GSDMD cleavage; this is the so-called noncanonical pathway pyroptosis [27]. Pyroptosis causes rapid plasma membrane rupture, resulting in the release of intracellular proinflammatory mediators IL-1*β*, IL-18, and HMGB1 [28]. In this paper, both mRNA and protein expressions of caspase1, NLRP3, ASC, and GSDMD in the KOA synovial tissue resulted in a significant upregulation compared with the Normal group. Meanwhile, by the depletion of macrophages with Clodronate Liposomes, the KOA+CL group showed a downregulation of these pyroptosis-related proteins compared with the KOA group. In addition, LPS+ATP-treated macrophages showed an increase in the number of pyroptotic cells and the protein expression of NLRP3, ASC, GSDMD, and GSDMD-N compared with the Normal group; this trend could be reversed by siRNA caspase1. Evidence above all suggests that pyroptosis occurs in synovial macrophages of KOA.

IL-1*β* and IL-18 are not only the downstream products of pyroptosis but are also important promoters of inflammation. IL-1*β*, as well as HMGB1, acts as a “gate keeper” of inflammation and directly contributes to synovial inflammation. In order to observe the relationship between macrophage pyroptosis and synovitis, HE staining was performed. Caspase1-inhibitor-treated rats (KOA+AYC group) showed a more orderly arranged lining of synovial cells and less inflammatory cell infiltration. In addition, both mRNA and protein expressions of IL-1*β*, IL-18, and HMGB1 were decreased in the KOA+AYC group. They are not only indicators for synovitis but are also downstream evidence for pyroptosis. Therefore, we speculate that synovial macrophage pyroptosis could promote synovitis, further contributing to KOA progression.

The response to TGF-*β* is a key event in the onset of synovial fibrosis [29]. Xue et al. have proven that smad4 silencing can suppress chronic inflammation and fibrosis in joint tissues by inhibiting the TGF-*β*/Smad pathway and can play a positive role in the prevention and treatment of joint stiffness [30]. In addition, gene expressions of PLOD2, COL1A1, and TIMP1 were upregulated both in OA fibroblasts stimulated with TGF-*β* and in mice with TGF-*β*-induced fibrosis [31]. Accordingly, we first measured the transverse diameters of the right knees to quantify the degree of knee swelling in rats and performed Sirius Red staining to observe collagen deposition. Then, we measured both mRNA and protein expressions of TGF-*β*, PLOD2, COL1A1, and TIMP1 to evaluate fibrosis in synovium. The KOA+AYC group showed a downregulation of all these profibrotic substances compared with the KOA group. Subsequently, as synovial fibroblasts are the main effector cells of synovial fibrosis, we established a coculture model of macrophages and synovial fibroblasts. After coculture for 48 hours, both mRNA and protein expressions of TGF-*β*, PLOD2, COL1A1, and TIMP1 in synovial fibroblasts showed a significant upregulation in the KOA group compared with the Normal group and a downregulation compared with the siRNA GSDMD group. Therefore, we speculate that the inhibition of synovial macrophage pyroptosis could alleviate fibrosis.

The current study still has a few limitations. First, due to the limitation of experimental conditions, we failed to extract macrophages from synovial tissue directly. We have tried the method of sorting magnetic beads, but the number of synovial macrophages obtained is really small. Fortunately, the extraction of macrophages from the peritoneal exudates as an alternative has been supported by many researchers [32–34]. As in clinical studies, macrophages in the peripheral blood of KOA patients are often used as substitutes for synovial macrophages. Secondly, the sample size of experimental animals would be bigger if possible. Future studies will focus on the exploration of medicine monomers which could inhibit pyroptosis in order to alleviate synovitis and fibrosis in KOA.

## 5. Conclusion

In summary, although a few limitations exist, this study still has demonstrated that the pyroptosis of synovial macrophages occurs in the development of KOA rats, which partly explains the pathogenesis of KOA. In addition, the study also indicates that synovial macrophages could be an effective target for the treatment of synovitis and fibrosis in knee osteoarthritis. The inhibition of synovial macrophage pyroptosis alleviates synovitis and fibrosis in KOA; therefore, it provides a new target for the clinical treatment of KOA.

## Figures and Tables

**Table 1:** Nucleotide sequences of primers used for RT-PCR amplification.

Target gene	Forward primer	Reverse primer
Caspase1	5′-TCGGAGAGTCGGAGCTGATGTT-3′	5′-CTCTGGGCAGGCAGCAAATTCT-3′
NLRP3	5′-CCAGACCTCCAAGACCACGACT-3′	5′-CCATCCGCAGCCAATGAACAGA-3′
ASC	5′-GAGTCTGGAGCTGTGGCTACTG-3′	5′-ATGAGTGCTTGCCTGTGTTGGT-3′
GSDMD	5′-TGCTTGCCGTACTCCATTCCATC-3′	5′-AGTTCTGAAGAGCCTGCCTCCA-3′
IL-1*β*	5′-CTCATTGTGGCTGTGGAGAAG-3′	5′-ACACTAGCAGGTCGTCATCAT-3′
IL-18	5′-ACCGCAGTAATACGGAGCAT-3′	5′-TCTGGGATTCGTTGGCTGTT-3′
HMGB1	5′-AGTTCAAGGACCCCAATGCC-3′	5′-GTCATCCGCAGCAGTGTTGTT-3′
TGF-*β*	5′-GACTCTCCACCTGCAAGACC-3′	5′-GGACTGGCGAGCCTTAGTTT-3′
COL1A1	5′-GTACATCAGCCCAAACCCCA-3′	5′-CAGGATCGGAACCTTCGCTT-3′
PLOD2	5′-AAGACGCTGCGATCAGAGAT-3′	5′-AGTGGGGGAGTCTTTTTCCC-3′
TIMP1	5′-TGTGGGAAATGCCACAGGTT-3′	5′-TTCCGTTCCTTAAACGGCCC-3′
GAPDH	5′-GGCCTTCCGTGTTCCTACC-3′	5′-ACTCGACACCTGCCCTCA-3′

## Data Availability

The data used to support the findings of this study are available from the corresponding author upon request.

## Abbreviations

KOA: Knee osteoarthritis
FLS: Fibroblast-like synoviocytes
NLRP: Nod-like receptor protein
ASC: Apoptosis-associated speck-like protein with a caspase-recruitment domain
GSDMD: Gasdermin D
LPS: Lipopolysaccharide
ATP: Adenosine triphosphate
siRNAs: Small interfering RNAs
HIF-1*α*: Hypoxia-inducible factors-1*α*
TGF-*β*: Transforming growth factor-*β*
PLOD2: Procollagen-lysine, 2-oxoglutarate 5-dioxygenase 2
COL1A1: Collagen type I *α*1 chain
TIMP1: Tissue inhibitor of metalloproteinase 1.
